# Successful perioperative management with damage control surgery following cardiac arrest due to massive postpartum hemorrhage: A case report

**DOI:** 10.1097/MD.0000000000035450

**Published:** 2023-09-29

**Authors:** Chan Hee Park, Jin Gon Bae, Jeong Woo Lee

**Affiliations:** a Department of Surgery, School of Medicine, Keimyung University Dongsan Medical Center, Daegu, Korea; b Department of Obstetrics and Gynecology, School of Medicine, Keimyung University Dongsan Medical Center, Daegu, Korea.

**Keywords:** cardiopulmonary resuscitation, damage control surgery, disseminated intravascular coagulation, hypovolemic shock, postpartum hemorrhage

## Abstract

**Introduction:**

Although declining, maternal mortality due to postpartum hemorrhage (PPH) remains significant. Here we report the case of a 31-year-old primipara patient admitted with cardiac arrest due to PPH.

**Case presentation:**

Labor was induced at gestational week 39, and the infant was delivered rapidly. Cardiac arrest due to PPH occurred during the transfer to our hospital, and the patient underwent cardiopulmonary resuscitation upon arrival to the emergency room. On admission, her hemoglobin level was 0.7 g/dL and she was in hypovolemic shock. Resuscitation and hysterectomy were performed immediately, including damage control surgery and gauze packing, to control the diffuse oozing bleeding due to severe disseminated intravascular coagulation. Relaparotomy for hemostasis was subsequently performed because of a decrease in hemoglobin level and blood pressure, and gauze packing was reinserted with temporary abdominal closure. Two days later, the abdominal wall was closed after confirming the absence of bleeding and the patient recovered well without further intervention.

**Conclusion:**

A prompt and assertive intensive response through collaborative efforts, utilizing feasible damage control surgery, can elegantly salvage uncontrolled bleeding in PPH patients with disseminated intravascular coagulation.

## 1. Introduction

Postpartum hemorrhage (PPH) is defined as blood loss >500 mL following vaginal delivery or >1 L after cesarean section, generally occurring within 24 hours of delivery.^[1]^ Every year, approximately 14,000,000 women worldwide experience PPH, of which severe PPH cases accounts for approximately 2%.^[2,3]^ Although the incidence of PPH is relatively low (5–10%), it remains a leading cause of pregnancy-related deaths in both developed and non-developed countries.^[4,5]^ The worldwide maternal mortality rate is decreasing, as is the maternal mortality rate in Korea, with a continuous decline rate of 2.3% per year.^[5]^

Risk factors for PPH include multiple pregnancies, surgical delivery, and chorioamnionitis. As PPH may occur in patients without known risk factors, bleeding may be difficult to predict.^[1]^ The common causes of PPH include bleeding in the placental implantation area following uterine atony, retained placenta, genital lacerations, uterine lacerations, and coagulopathy. Uterine atony accounts for most causes of primary PPH.^[6]^

Herein, we present a case of severe PPH treated with surgical gauze packing as a damage control surgery (DCS) following cardiac arrest due to hypovolemic shock and failed conventional treatment.

## 2. Case presentation

A 31-year-old primipara with PPH was admitted to our hospital with cardiac arrest secondary to hypovolemic shock. The patient had no remarkable medical history, reported, or specific medical history during pregnancy. In the 39th week of pregnancy, the patient underwent labor induction at a local clinic, which was unsuccessful on the first day of pregnancy. On the second day, labor induction was repeated, and she felt colic and delivered rapidly within an hour. A female infant weighing 2.2 kg in relatively stable condition. However, the patient continued to bleed immediately after vaginal delivery and was admitted to our hospital 2 hours after delivery because of decreased blood pressure (BP) and loss of consciousness. Cardiac arrest occurred during transfer from the local clinic to our hospital, and she was admitted to the emergency room (ER) while undergoing cardiopulmonary resuscitation (CPR).

On admission to the ER, consciousness was not confirmed, and the patient’s pupils were fully dilated. A large volume of vaginal bleeding was observed and the pads were soaked in blood. The BP and heart rate were not measured. After 3 cycles of CPR, spontaneous circulation was restored, and a BP of 48/30 mm Hg and heart rate of 116 beats/min were recorded. The hemoglobin (Hb) level was 0.7 g/dL at admission to the ER. Other laboratory data included a platelet count of 21.00 × 10^3^ cells/μL, prothrombin time > 80 s, activated partial thromboplastin time > 200 s, and fibrinogen level < 40 mg/dL. Arterial blood gas analysis revealed a pH of 6.993, PaO_2_ level of 17.6 mm Hg, PaCO_2_ level of 36.3 mm Hg, HCO_3_ level of 8.8 mmol/L, and lactic acid level of 11.3 mmol/L.

At the time of admission to the ER, transfusion of 4 units of packed red blood cells brought from the local clinic with the patient was initiated. The patient remained unstable from the time of admission to the ER until transfer to the operating room. Exploratory laparotomy was performed 1 hour after arrival at the ER, but imaging evaluation could not be performed. The surgical findings were as follows: a large uterus without signs of uterine contraction, right anterior cervical laceration, and prolonged lower uterine segment. Therefore, the bleeding was controlled, and cesarean hysterectomy with right salpingo-oophorectomy and internal iliac artery ligation were performed. Furthermore, DCS (gauze packing and temporary abdominal wall closure) was performed because of severe diffuse oozing bleeding caused by dilutional coagulopathy and disseminated intravascular coagulation (DIC).

The patient’s vital signs remained unstable even after surgery, and progression was closely monitored during resuscitation in the intensive care unit. The blood transfused on the first day after hospital admission comprised 24 units of packed red blood cells, 16 units of fresh frozen plasma, and 12 units of platelets. However, active dye leakage was not observed on computed tomography because of continuous oozing bleeding and a decrease in Hb level, and a large hematoma was observed bilaterally around the internal iliac vessels and pelvis. Arterial angiography was performed to identify the bleeding focus, but no evidence of active bleeding was found. Additional procedures such as arterial embolization were not performed. An exploratory laparotomy was subsequently performed to identify venous bleeding and any hidden bleeding foci. Careful hemostasis and suture ligation were performed around both iliac vessels, oozing the bleeding sites in the previously sutured vaginal wall and multiple bleeding sites in the pelvic cavity area. Additionally, gauze packing and temporary abdominal wall closure were performed. Repeat exploratory laparotomy was performed 2 days after stabilization, revealing no further bleeding. The abdominal wall was successfully closed in layers. After this treatment, the Hb level remained unchanged and there was no evidence of bleeding. The patient was transferred to the general ward for conservative treatment after successful weaning from a ventilator in the intensive care unit. The surgical wound was clean with no signs of surgical site infection. There were no specific complaints, except for discomfort in the sternum area, which is a post-CPR symptom.

## 3. Discussion

Approximately 0.1% of all pregnant women experience PPH with severe DIC.^[7]^ If hemostasis fails, even in places where it generally works well and there are signs of intravenous line oozing bleeding, DIC should be suspected before laboratory test results are obtained. Dilutional coagulopathy and DIC develop in most cases because of large volumes of fluid infusion and blood transfusion for the treatment of unstable patients following massive bleeding during or after delivery.^[8]^ To reduce the incidence of DIC in patients with PPH, transfusion ratios of 1:1:1 or 2:1:1 for RBC, fresh frozen plasma, and platelets are recommended instead of crystalloid or colloid use in patients with massive bleeding.^[9]^ In the present case, cardiac arrest due to hypovolemic shock would not have occurred if transfusion were performed more promptly when massive bleeding was predicted after delivery. We conformed to the transfusion ratio (1:1:1 or 2:1:1) to the greatest extent possible by using a massive transfusion protocol to reduce the effects of DIC and dilutional coagulopathy. As a result, rapid correction of coagulopathy and prompt control of bleeding were achieved.

Although the treatment protocol for PPH differs depending on the institution or medical staff, uterine preservation is prioritized according to several guidelines.^[10,11]^ Uterine massage with the use of uterine contractors such as oxytocin, prostaglandins, and primary sutures may be applied in cases of birth canal laceration. Intrauterine packing, ballooning, and angio-embolization were performed. However, if there is no response to conservative treatment that attempts to stop bleeding while preserving the uterus, hysterectomy should be promptly performed as the last option for curative treatment. Compared with conservative treatment, loss of fertility and various complications caused by hysterectomy lead to hesitation; however, delaying treatment decisions may increase the patient’s risk of deterioration to multi-organ failure. However, in patients with prominent massive bleeding immediately after delivery and who tend to experience cardiac arrest due to hypovolemic shock, such as our patient, there is insufficient time for conservative treatment. Therefore, we opted for curative treatment of PPH as the best life-saving option, performing internal iliac artery ligation and emergent hysterectomy.

DCS is a surgical method predominantly used for trauma. In the present case, it was successfully applied as a stepwise surgical method to treat a deteriorating general condition due to massive bleeding with an increased bleeding tendency due to coagulopathy.^[12]^ DCS can be applied to avoid extensive surgery and stabilize patients in an unstable state, allowing staged surgery following successful initial resuscitation.^[13]^ Lethal triads, such as hypothermia, coagulopathy, and metabolic acidosis, exacerbate each other, sometimes leading to massive bleeding and death.^[14]^ DCS enables definitive surgery after correction of lethal triads, eventually achieving a better prognosis. In our patient, gauze packing and temporary abdominal closure were performed for diffuse oozing bleeding caused by an increased bleeding tendency due to exacerbated coagulopathy. DCS was performed after correcting the coagulation factor, followed by definitive surgery. This was the best treatment option for the patient.

In conclusion, uncontrolled oozing bleeding may occur in PPH patients with DIC, and DCS may be feasible after consultation with surgeons and obstetricians. With the collaborative efforts of surgical intensivists, a prompt and assertive intensive response can effectively salvage patients with PPH, thereby circumventing grave complications including DIC and multiple organ failure.

## Author contributions

**Conceptualization:** Chan Hee Park, Jin Gon Bae, Jeong Woo Lee.

**Data curation:** Chan Hee Park.

**Formal analysis:** Chan Hee Park.

**Investigation:** Chan Hee Park.

**Resources:** Jin Gon Bae.

**Visualization:** Jeong Woo Lee.

**Writing – original draft:** Chan Hee Park.

**Writing – review & editing:** Chan Hee Park.
